# Protein Phosphatase 2A in Lipopolysaccharide-Induced Cyclooxygenase-2 Expression in Murine Lymphatic Endothelial Cells

**DOI:** 10.1371/journal.pone.0137177

**Published:** 2015-08-28

**Authors:** Yu-Fan Chuang, Mei-Chieh Chen, Shiu-Wen Huang, Ya-Fen Hsu, George Ou, Yu-Jou Tsai, Ming-Jen Hsu

**Affiliations:** 1 Graduate Institute of Medical Sciences, College of Medicine, Taipei Medical University, Taipei, Taiwan; 2 Department of Microbiology and Immunology, College of Medicine, Taipei Medical University, Taipei, Taiwan; 3 Graduate Institute of Pharmacology, College of Medicine, National Taiwan University, Taipei, Taiwan; 4 Division of General Surgery, Department of Surgery, Landseed Hospital, Taoyuan, Taiwan; 5 Department of Medicine, University of British Columbia, Vancouver, British Columbia, Canada; 6 Department of Internal Medicine, Yuan's General Hospital, Kaohsiung, Taiwan; 7 Department of Pharmacology, School of Medicine, College of Medicine, Taipei Medical University, Taipei, Taiwan; Medical College of Wisconsin, UNITED STATES

## Abstract

The lymphatic endothelium plays an important role in the maintenance of tissue fluid homeostasis. It also participates in the pathogenesis of several inflammatory diseases. However, little is known about the underlying mechanisms by which lymphatic endothelial cell responds to inflammatory stimuli. In this study, we explored the mechanisms by which lipopolysaccharide (LPS) induces cyclooxygenase (COX)-2 expression in murine lymphatic endothelial cells (SV-LECs). LPS caused increases in *cox-2 mRNA* and protein levels, as well as in COX-2 promoter luciferase activity in SV-LECs. These actions were associated with protein phosphatase 2A (PP2A), apoptosis signal-regulating kinase 1 (ASK1), JNK1/2 and p38MAPK activation, and NF-κB subunit p65 and C/EBPβ phosphorylation. PP2A-ASK1 signaling blockade reduced LPS-induced JNK1/2, p38MAPK, p65 and C/EBPβ phosphorylation. Transfection with PP2A siRNA reduced LPS’s effects on p65 and C/EBPβ binding to the *COX-2* promoter region. Transfected with the NF-κB or C/EBPβ site deletion of COX-2 reporter construct also abrogated LPS’s enhancing effect on COX-2 promoter luciferase activity in SV-LECs. Taken together, the induction of COX-2 in SV-LECs exposed to LPS may involve PP2A-ASK1-JNK and/or p38MAPK-NF-κB and/or C/EBPβ cascade.

## Introduction

Lymphatic vessels (LVs) are present in most vascularized tissues, and transport fluids, soluble antigens and immune cells. It is believed that lymphatic vasculature not only contributes to tissue fluid homeostasis [1, 2], but also plays a critical role in modulating inflammatory and immune processes [3]. Recent studies demonstrated that peripheral lymphatic vasculature undergoes substantial changes under pathologic conditions [1, 2]. Lymphatic vascular network expansion has been observed in human inflammatory diseases [4] and in experimental inflammatory mouse models of arthritis [5], dermatitis [6] and inflammatory bowel disease [7]. However, the underlying mechanisms by which lymphatic endothelium responds to inflammatory stimuli remain to be fully defined. Lipopolysaccharide (LPS), the major Gram-negative bacteria cell wall component, elicits most of the clinical manifestations of bacterial infection [8–10]. LPS-elicited inflammatory process is attributed to the expression of cyclooxygenase (COX)-2, which is a key enzyme in prostaglandin biosynthesis and plays a crucial role in inflammation-associated diseases [11]. COX-2 was shown to regulate vascular endothelial functions [12]. However, little is known about how LPS regulates COX-2 expression in lymphatic endothelial cells (LECs).

Transcription factor NF-κB plays an important role in regulating pro-inflammatory gene expression. The aberrant activation of NF-κB has been implicated in the pathogenesis of inflammatory disorders [13]. NF-κB contributes to COX-2 expression in response to pro-inflammatory stimuli such as LPS [14]. In addition to NF-κB, the 5’-flanking region of the *cox-2* gene contains many other transcription factor-binding sites. These transcription factors include C/EBP, CREB and SP1 [15–18]. Among these, activation of C/EBPβ also plays an important regulatory role in COX-2 induction [17–20]. We demonstrated previously that NF-κB and C/EBPβ activation contributes to LPS-induced COX-2 expression in vascular endothelial cells [14]. However, the roles of NF-κB and C/EBPβ in regulating COX-2 expression in LECs remain incompletely understood.

Reversible protein phosphorylation catalyzed by protein kinases and protein phosphatases regulates various cellular processes [21]. The activation of mitogen-activated protein kinases (MAPKs) contributes to cellular responses in the presence of inflammatory stimuli [22, 23]. Apoptosis signal-regulating kinase 1 (ASK1) is a critical upstream activator of p38MAPK and JNK1/2 [24, 25]. ASK1 plays an essential role in various cellular responses including apoptosis, cell survival, differentiation, and production of inflammatory cytokines [24, 26–28]. In addition to protein kinases, protein phosphatases may be involved in COX-2 expression as well. Recent studies have highlighted a pivotal role of serine/threonine protein phosphatases such as protein phosphatase 2A (PP2A) in modulating inflammatory responses [29, 30]. PP2A was reported to activate p38MAPK or JNK1/2 via ASK1 [30, 31]. However, little information is available about the role of PP2A in regulating ASK1 signaling and subsequent COX-2 expression in LECs exposed to LPS. We therefore attempted to establish the causal role of PP2A in LPS-induced COX-2 expression in murine lymphatic endothelial cells (SV-LECs). In this study, we demonstrated that LPS induces PP2A activation, which results in the activation of ASK1, p38MAPK and JNK1/2, and subsequent binding of p65 and C/EBPβ to the *cox*-2 promoter region; together, these culminate in the increased COX-2 expression in LPS-stimulated SV-LECs.

## Materials and Methods

### Reagents

Lipopolysaccharides (LPS) purified by phenol extraction from Escherichia coli 0127:B8 and 3-(4,5-dimethylthiazol-2-yl)-2,5-diphenyl tetrazolium bromide (MTT) was purchased from Sigma-Aldrich (St. Louis, MO, USA). DMEM, optiMEM, fetal bovine serum (FBS), penicillin, and streptomycin were purchased from Invitrogen (Carlsbad, CA, USA). Okadaic acid, p38MAPK inhibitor III, JNK inhibitor II, U0126, Turbofect in vitro transfection reagent and antibody specific for COX-2 were purchased from Merck Millipore (Billerica, MA, USA). Normal IgG, antibodies specific for p65, C/EBPβ and PP2A catalytic subunit (PP2A-C) were purchased from Santa Cruz Biotechnology (Santa Cruz, CA, USA). Antibodies specific for α-tubulin, p-p65 Ser536, JNK2, p38MAPK and anti-mouse or anti-rabbit immunoglobulin G (IgG)-conjugated horseradish peroxidase (HRP) antibodies were purchased from GeneTex Inc (Irvine, CA, USA). Antibodies specific for p-p38MAPK, p-JNK1/2, p-ERK1/2, ERK1/2, p-C/EBPβ and IκBα were purchased from Cell Signaling Technology (Beverly, MA, USA). The HA-tagged expression constructs for catalytically inactive ASK1-K709E [ASK1 dominant-negative mutant (DN)] and pcDNA were derived as described previously [32]. Murine COX-2 promoter with wild type construct (native _966/+23) and mutant constructs cloned into pGL3-basic vector (Promega, Madison, WI, USA) were kindly provided by Dr. Byron Wingerd (Michigan State University, East Lansing, MI). C/EBP reporter construct, p/T81 C/EBP-luc, was kindly provided by Dr. Kjetil Tasken (University of Oslo, Oslo, Norway). NF-κB-Luc, Renilla-luc and Dual-Glo luciferase assay system were purchased from Promega (Madison, WI, USA). All materials for immunoblotting were purchased from Invitrogen (Carlsbad, CA, USA). All other chemicals were obtained from Sigma (St. Louis, MO, USA).

### Cell culture

The mouse LEC line SV-LEC was kindly provided by Dr. J.S. Alexander (Shreveport, LA, USA), and was cultured as previously described [33, 34]. The lymphatic endothelial markers such as vascular endothelial growth factor receptor 3 (VEGFR-3, Flt-4), lymphatic vessel endothelial hyaluronan receptor (LYVE-1) and prospero-related homeobox 1 (Prox1) were used to confirm it maintains LEC phenotype *in vitro* (S1 Fig).

### Immunoblot analysis

Immunoblot analyses were performed as described previously [34]. Briefly, cells were lysed in extraction buffer containing 10 mM Tris (pH 7.0), 140 mM NaCl, 2 mM PMSF, 5 mM DTT, 0.5% NP-40, 0.05 mM pepstatin A, and 0.2 mM leupeptin. Samples of equal amounts of protein were subjected to sodium dodecylsulfate polyacrylamide gel electrophoreses (SDS-PAGE) and transferred onto a nitrocellulose membrane which was then incubated in TBST buffer (150 mM NaCl, 20 mM Tris-HCl, and 0.02% Tween 20; pH 7.4) containing 5% non-fat milk. Proteins were visualized by specific primary antibodies and then incubated with horseradish peroxidase-conjugated secondary antibodies. Immunoreactivity was detected based on enhanced chemiluminescence per the instructions of the manufacturer. Quantitative data were obtained using a computing densitometer with a scientific imaging system (Kodak, Rochester, NY, USA).

### Cell viability assay (MTT assay)

Cell viability was measured by the colorimetric 3-(4,5-dimethylthiazol-2-yl)-2,5-diphenyl tetrazolium bromide (MTT) assay as described previously [14].

### Lactate dehydrogenase (LDH) release assay

LDH leakage was measured to quantify cytotoxicity with a CytoTox96 non-radioactive cytotoxicity assay kit (Promega, Madison, WI, USA) as described previously [35]

### Transfection in SV-LECs and dual luciferase reporter assay

SV-LECs (7 X 10^4^ cells per well) were transfected with COX-2-luc (1 μg), mNFκB-COX-2-luc (1 μg), mC/EBPβ-COX-2-luc (1 μg), NF-κB-luc (1 μg), or C/EBPβ-luc (1 μg) plus Renilla-luc (0.25 μg) using Turbofect transfection reagent (Upstate Biotechnology, Lake Placid, NY, USA) for 48 h. After transfection, cells with or without treatments were harvested. The luciferase activity was then determined using a Dual-Glo luciferase assay system kit (Promega, Madison, WI, USA) according to manufacturer’s instructions, and was normalized on the basis of Renilla luciferase activity. SV-LECs were also transfected with pcDNA (1 μg) or ASK1DN (1 μg) using Turbofect transfection reagent (Upstate Biotechnology, Lake Placid, NY, USA) for 48 h. After transfection, cells with or without treatments were harvested for immunoblotting.

### Reverse-transcription polymerase chain reaction (RT-PCR)

Total RNA was isolated from cells using the RNAspin RNA isolation kit (GE Healthcare, Little Chalfont, UK). The RT-PCR was then conducted following the manufacturer’s instructions (Super Script On-Step RT-PCR system, Invitrogen). Primers used for amplification of the COX-2, VEGFR3, LYVE-1, Prox-1 and GAPDH fragments were as follows: COX-2, sense 5'-CCCCCACAGTCAAAGACACT-3' and antisense 5'-CTCATCACCCCACTCAGGAT-3'; VEGFR3, sense 5'-ACATCCAGCTGTACC CCAAG-3' and antisense 5'-gagccactcgacactgatga-3'; LYVE-1, sense 5'- gctgatgacgtcaacgctaa-3' and antisense 5'-acctggaagcctgtctctga-3'; Prox-1, sense 5'-gcacgtgagctatgg agtga-3' and antisense 5'-tcacagagacagcaggttgg-3'; GAPDH, sense 5’-CCTTCATTGACCTCAACTAC-3’ and antisense 5’-GGAAGGCCATGCCAGTGAGC-3’. GAPDH was used as the internal control. The PCR was performed with the following conditions: a 5-min denaturation step at 94°C, 30 cycles of a 30-s denaturation step at 94°C, a 30-s annealing step at 56°C, and a 45-s extension step at 72°C to amplify COX-2, VEGFR3, LYVE-1, Prox-1 and GAPDH cDNA. The amplified fragment sizes for COX-2, VEGFR3, LYVE-1, Prox-1 and GAPDH were 191, 323, 386, 364 and 594 bp, respectively. PCR products were run on an agarose gel, stained with ethidium bromide, and visualized by ultraviolet illumination.

### Chromatin immunoprecipitation (ChIP) assay

A ChIP assay was performed as described previously [34]. Briefly, cells were cross-linked with 1% formaldehyde at 37°C for 10 min and then rinsed with ice-cold PBS. Cells were then harvested in SDS lysis buffer, sonicated six times for 15 s each, and then centrifuged for 10 min. Supernatants were collected and diluted in ChIP dilution buffer, followed by immunoclearing with gentle rotation with 80 μl protein A-agarose slurry for 1 h at 4°C. An aliquot of each sample was used as “input” in the PCR analysis. The remainder of the soluble chromatin was incubated at 4°C overnight with p65 and C/EBPβ antibodies or control IgG (Santa Cruz Biotechnology). Immune complexes were collected by incubation with 60 μl protein A-Magnetic Beads (Millipore, Billerica, MA, USA) for 2 h at 4°C with gentle rotation. The complexes were washed sequentially for 5 min in the following three washing buffers: low-salt immune complex washing buffer, high-salt immune complex washing buffer, and LiCl immune complex washing buffer. Precipitates were washed twice with Tris-EDTA buffer. The complexes were then eluted twice with two 100 μl aliquots of elution buffer. The cross-linked chromatin complex was reversed in the presence of 0.2 M NaCl and heating at 65°C for 4 h. DNA was purified using GP DNA purification spin columns (Viogene, New Taipei City, Taiwan). PCR was performed using PCR MasterMix (Promega, Madison, WI, USA), according to the manufacturer’s protocol. Ten percent of the total purified DNA was used for the PCR in 50 μl reaction mixture. The 163-bp and 173-bp COX-2 promoter fragments between -466 and -395 (for p65 binding sequence), and -138 and -85 (for C/EBP binding sequence) were amplified using the primer pairs, for p65 binding sequence, sense: 5′- gta gct gtg tgc gtg ctc tg -3′ and antisense: 5′- ctc cgg ttt cct ccc agt -3′; for C/EBP binding sequence, sense: 5′- agc tct ctt ggc acc acc t -3′ and antisense: 5′- acg tag tgg tga ctc tgt ctt tcc gc -3′, in 30 cycles of PCR. This was done: at 95°C for 30 s, at 56°C for 30 s, and at 72°C for 45 s. The PCR products were analyzed by 1.5% agarose gel electrophoresis.

### PP2A activity assay

A Serine/Threonine phosphatase assay system (Promega, Madison, WI, USA) was used to measure phosphate release as an index of phosphatase activity according to the manufacturer's instructions with modifications. Briefly, 200 μg of cellular proteins were incubated for 2 h at 4°C with 2 μg anti-PP2A-C antibody (GeneTex, Irvine, CA, USA), and 20 μl protein A-Magnetic Beads (Millipore, Billerica, MA, USA), to immunoprecipitate PP2A-C. Immune complexes were then collected, washed three times, and incubated with phosphoprotein, the substrate (amino acid sequence RRApTVA, 100 μM), in protein phosphatase assay buffer (20 mM 4-morpholinepropanesulfonic acid (pH 7.5), 60 mM 2-mercaptoethanol, 0.1 M NaCl, and 0.1 mg/ml serum albumin). Reactions were initiated by the addition of the phosphoprotein substrate and carried out for 15 min at 37°C. We also prepared appropriate phosphate standard solutions containing free phosphate for standard curve. Reactions were terminated by the addition of 50 μl of the Molybdate Dye solution. The absorbance at 600 nm was measured on a microplate reader. Nonspecific hydrolysis of RRApTVA by lysates was assessed in normal IgG immunoprecipitates.

### Statistical analysis

Results are presented as the mean ± S.E. from at least three independent experiments. One-way analysis of variance (ANOVA) followed by, when appropriate, the Newman-Keuls test was used to determine the statistical significance of the difference between means. A *p* value of < 0.05 was considered statistically significant.

## Results

### LPS induced COX-2 expression in SV-LECs

**Fig 1:**
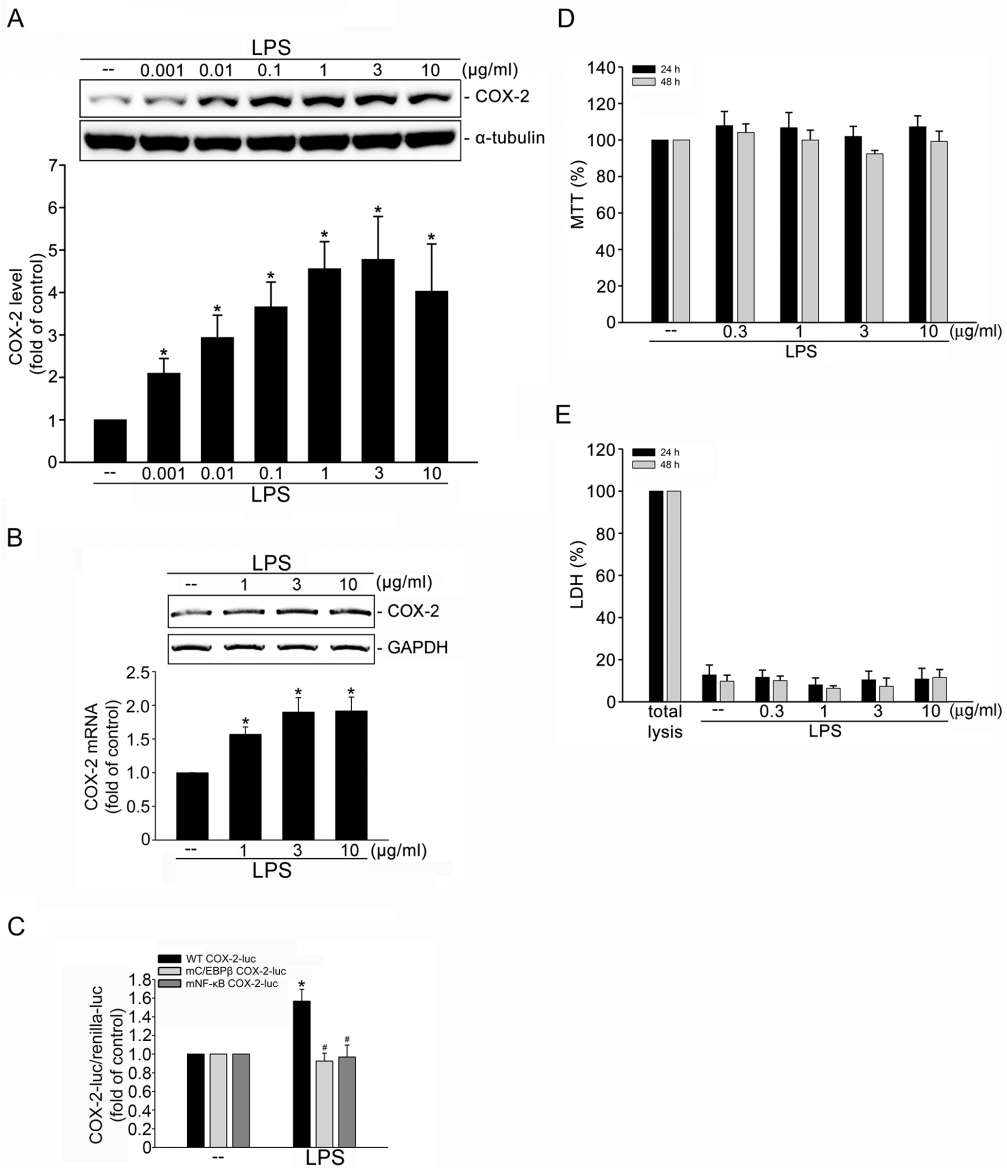
LPS induced COX-2 expression in SV-LECs. Cells were treated with vehicle or LPS at the indicated concentrations for 24 h. After treatment, cells were harvested to assess the COX-2 level by immunoblotting. Each column represents the mean ± S.E.M. of five independent experiments. * p<0.05, compared with the control group. **B.** Cells were treated with vehicle or LPS at the indicated concentrations for 6 h. After treatment, the extent of *cox-2* mRNA was determined by an RT-PCR assay as described in the “Materials and methods” section. Each column represents the mean ± SEM of six independent experiments. * p<0.05, compared with the control group. **C.** Cells were transiently transfected with WT COX-2-luc (wild type), mC/EBP COX-2-luc (C/EBP site mutant) or mNFκB COX-2-luc (NFκB site mutant) and Renilla-luc for 48 h. Luciferase activity was then determined after treatment of LPS (3 μg/ml) for another 24 h as described in the “Materials and methods” section. Data represent the mean ± S.E.M. of five independent experiments performed in duplicate. * p<0.05, compared with the vehicle-treated group; ^#^ p<0.05, compared with the LPS-treated cells transfected with WT COX-2-luc. **D.** SV-LECs were treated with indicated concentrations of LPS for 24 or 48 h. Cell viability was determined by MTT assay. Each column represents the mean ± S.E.M. of five independent experiments performed in triplicate **E.** SV-LECs were treated with indicated concentrations of LPS for 24 or 48 h. The cytotoxicity of LPS was determined by LDH assay. Cells were also treated with cell lysis buffer (total lysis, TL) to serve as positive control. Each column represents the mean ± S.E.M. of five independent experiments performed in triplicate.

We used an immunoblotting analysis to examine the COX-2 levels in SV-LECs exposed to LPS. Treatment with LPS (0.001–10 μg/ml) over 24 h led to increases in COX-2 protein levels in SV-LECs in a concentration-dependent manner (Fig 1A). The maximum effect of LPS in COX-2 induction was observed at doses ranging from 1 to 10 μg/ml. The high concentrations (1–3 μg/ml) of LPS were thus selected to explore the signaling cascades involved in COX-2 induction in SV-LECs in the following experiments. LPS also significantly increased *cox-2 mRNA* levels in SV-LECs after 6 h exposure to LPS (1–10 μg/ml) (Fig 1B), which confirms that the increase in protein level is a result of increased transcription. It is conceivable that LPS activates transcription factors, leading to COX-2 expression in SV-LECs. The 5’-flanking region of the murine *cox-2* gene contains many consensus sequences, including those for CCAAT/enhancer-binding protein (C/EBPß) and NF-κB. C/EBPß and NF-κB have been reported to contribute to COX-2 elevation in different types of cells in response to various stimuli [15–18]. To examine the causal roles of C/EBPβ and NF-κB in COX-2 induction in LPS-stimulated SV-LECs, the wild-type murine COX-2 reporter construct (-966/-23, WT COX-2-luc) and mutant reporter constructs with either a C/EBPβ (-138/-130, mC/EBPβ COX-2-luc) or a NF-κB (-402/-395, mNF-κB COX-2-luc) site deletion were separately transfected into SV-LECs. Our results demonstrated that LPS significantly increased COX-2 promoter luciferase activity in cells transfected with wild-type murine COX-2 reporter construct, but this effect was reduced in cells transfected with mutant constructs with C/EBPβ or NF-κB deletion (Fig 1C). Together, these suggest that the activation of C/EBPβ or NF-κB contributes to LPS-induced COX-2 expression in SV-LECs. To determine whether LPS affects cell viability in SV-LECs, a MTT assay was employed. As shown in Fig 1D, treatment of cells with LPS (0.3–10 μg/ml) for 24 or 48 h did not alter cell viability. Moreover, LPS did not significantly increase LDH release in SV-LECs after 24 or 48 h exposure to LPS (0.3–10 μg/ml) as determined by a LDH assay (Fig 1E). These results indicated that 48 h treatment of LPS at concentrations ranging from 0.3 to 10 μg/ml did not cause cell death in SV-LECs.

### LPS induced C/EBPβ and NF-κB activation in SV-LECs

**Fig 2:**
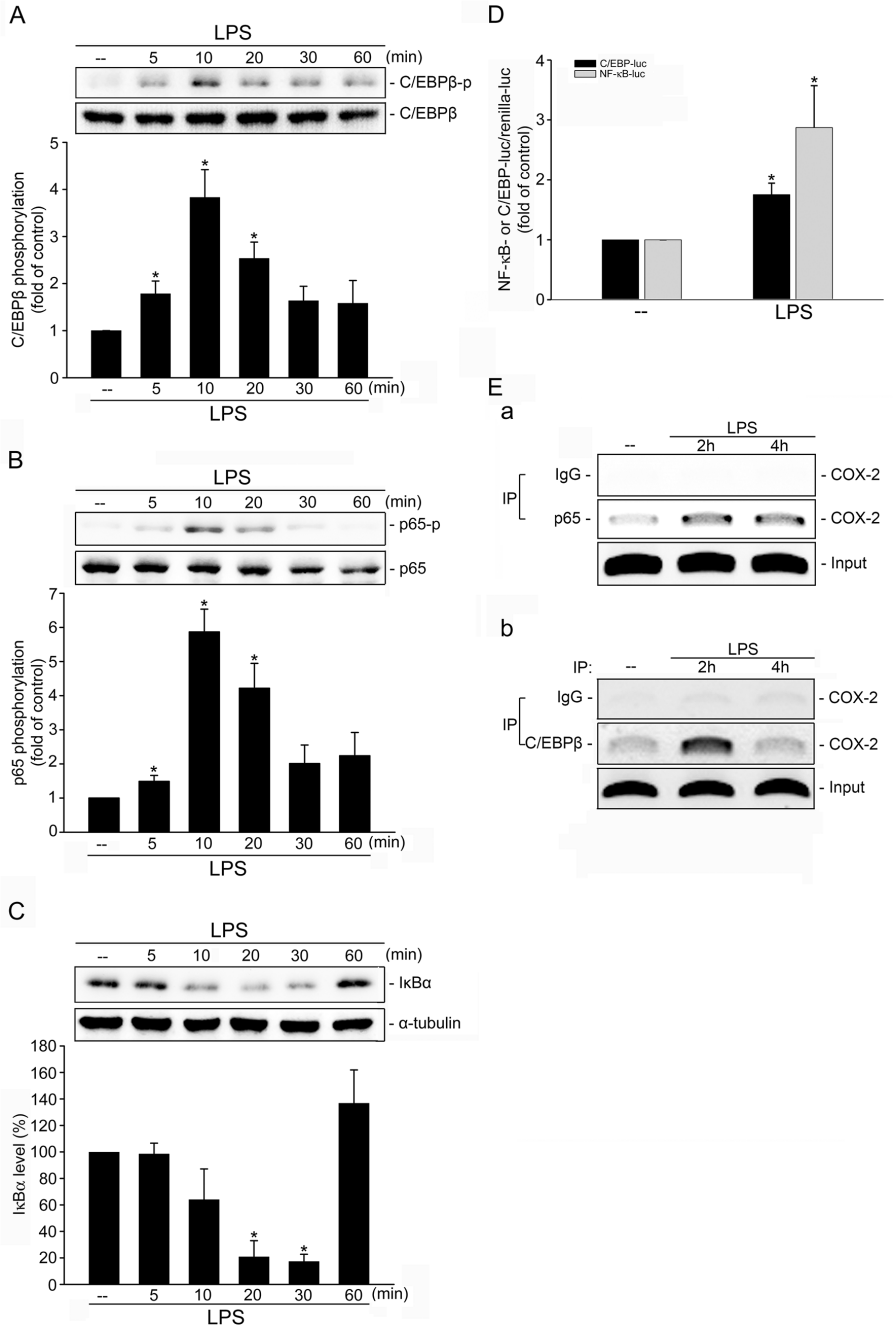
Involvement of C/EBPβ and NF-κB in LPS-induced COX-2 expression in SV-LECs. Cells were treated with LPS (3 μg/ml) for the indicated time periods. Cells were then harvested. The extent of C/EBPβ **(A)** or p65 **(B)** phosphorylation or IκBα level **(C)** was determined by immunoblotting. Compiled results are shown at the bottom of the chart. Each column represents the mean ± S.E.M. of at least six independent experiments. * p<0.05, compared with the control group. **D.** Cells were transiently transfected with C/EBP-luc or κB-luc and renilla-luc for 48 h. After transfection, cells were treated with LPS (3 μg/ml) for another 24 h. Luciferase activity was then determined as described in the “Materials and Methods” section. Data represent the mean ± S.E.M. of eight independent experiments performed in duplicate. * p<0.05, compared with the control group. **E.** Cells were incubated with LPS (3 μg/ml) for indicated time periods and the ChIP assay was performed as described in the ‘‘Materials and methods” section. Typical traces representative of three independent experiments with similar results are shown. IP: immunoprecipitation.

It has been reported that C/EBPß phosphorylation promotes its transcriptional activity [36]. In addition, NF-κB activity is tightly regulated by its binding to the inhibitory IκBα protein, which prevents cytosolic NF-κB from entering the nucleus, and the phosphorylation of its p65 subunit, which may account for its nuclear localization and transcriptional activity [37]. We therefore assessed the phosphorylation status of C/EBPß and p65 in SV-LECs exposed to LPS, which were both increased in time-dependent manner (Fig 2A and 2B). The IκBα protein also significantly decreased after exposure to LPS for 20 min, but returned to the baseline level after treatment for 1 h (Fig 2C). In keeping with these results, results from reporter assays demonstrated that LPS increased NFκB- and C/EBP-luciferase activities in SV-LECs (Fig 2D). A ChIP experiment was conducted to determine whether p65 or C/EBPß is recruited to the endogenous COX-2 promoter region in response to LPS. Primers encompassing the COX-2 promoter regions (-466 to -304 and -230 to -60) containing putative p65 and C/EBPß binding sites were used. As shown in Fig 2E, the binding of p65 to the COX-2 promoter region (-466/-304) increased, as did C/EBPß binding to the COX-2 promoter region (-230/-60) after LPS exposure. These results suggest that p65 and C/EBPß contributed to LPS-induced COX-2 expression in SV-LECs.

### MAPKs mediates LPS-induced COX-2 expression in SV-LECs

**Fig 3:**
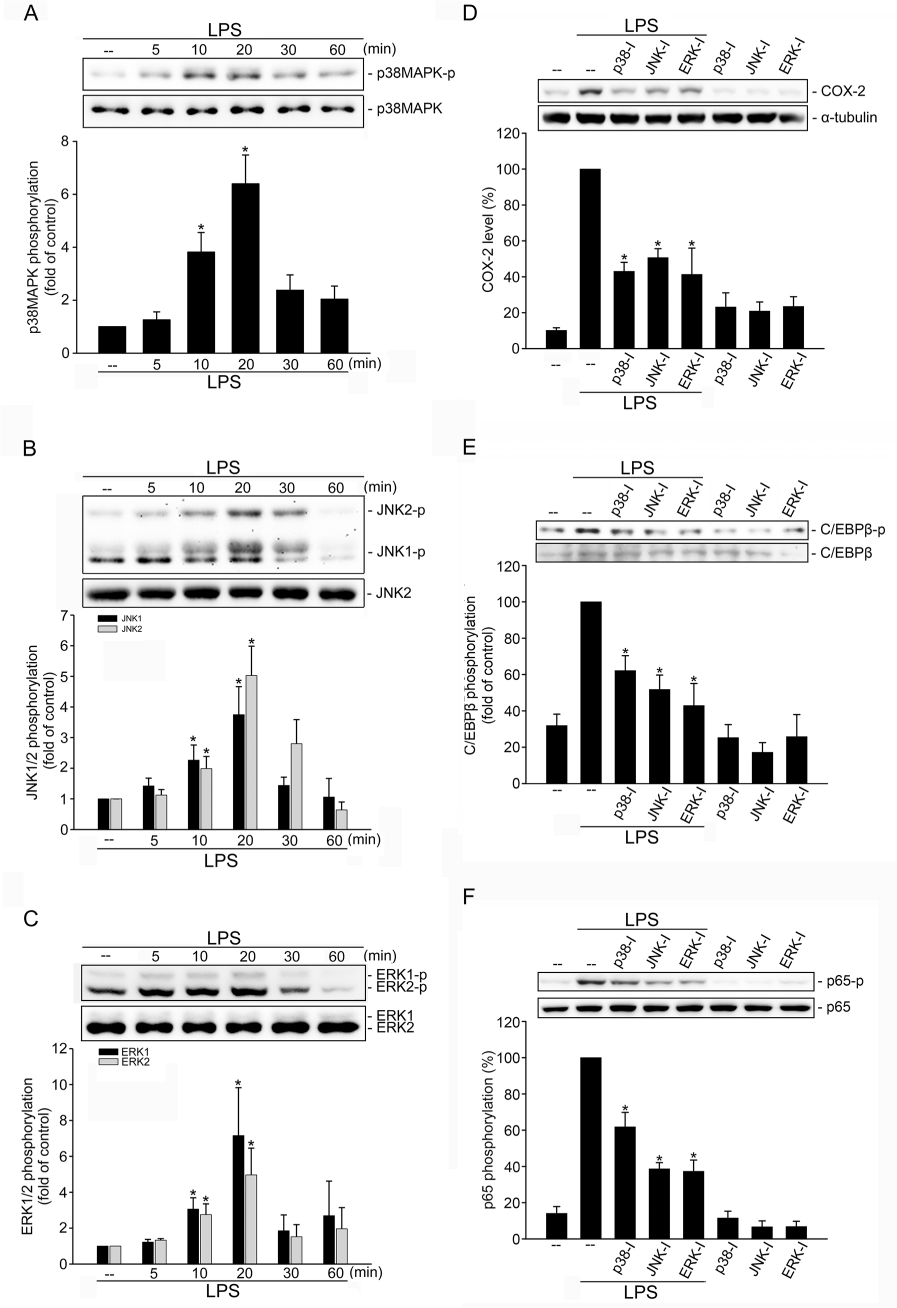
MAPKs contributed to LPS-induced C/EBPβ and NF-κB phosphorylation and COX-2 expression in SV-LECs. Cells were treated with LPS (3 μg/ml) for the indicated time periods. Cells were then harvested and p38MAPK **(A)**, JNK1/2 **(B)** or ERK1/2 **(C)** phosphorylation was determined by immunoblotting. Compiled results are shown at the bottom of the chart. Each column represents the mean ± S.E.M. of at least four independent experiments. * p<0.05, compared with the control group. Cells were pretreated with p38MAPK inhibitor III (a p38MAPK inhibitor, p38MAPK-I, 1 μM), JNK1/2 inhibitor II (a JNK1/2 inhibitor, JNK1/2-I, 5 μM) or U0126 **(**a ERK1/2 inhibitor, ERK1/2-I, 1 μM) for 30 min followed by treatment with LPS (3 μg/ml) for another 24 h **(D)** or 10 min (**E** and **F**). The COX-2 level **(D)** or C/EBPβ **(E)** and p65 **(F)** phosphorylation were then determined by immunoblotting. Compiled results are shown at the bottom of the chart. Each column represents the mean ± S.E.M. of at least four independent experiments. * p<0.05, compared with the group treated with LPS alone.

We next explored the signaling cascades that may contribute to LPS-induced COX-2 expression in SV-LECs. We examined whether p38MAPK, JNK1/2 and ERK1/2 phosphorylation are altered in SV-LECs after LPS exposure. As shown in Fig 3A, LPS caused an increase in p38MAPK phosphorylation in a time-dependent manner. JNK1/2 (Fig 3B) and ERK1/2 (Fig 3C) phosphorylation were also increased in cells exposed to LPS. In contrast, p38MAPK inhibitor III (p38-I) significantly suppressed LPS-induced COX-2 expression (Fig 3D). Similarly, JNK1/2 inhibitor II (JNK-I) and U0126 (an ERK signaling inhibitor, ERK-I) also significantly suppressed LPS-induced COX-2 expression (Fig 3D). Furthermore, LPS’s enhancing effects on C/EBPß (Fig 3E) and p65 (Fig 3F) phosphorylation were reduced in the presence of p38MAPK inhibitor III, JNK1/2 inhibitor II or U0126. Taken together, these results suggest the causal roles of MAPKs in C/EBPß and p65 activation and COX-2 induction in LPS-stimulated SV-LECs.

### ASK1 contributes to LPS-induced COX-2 expression in SV-LECs

**Fig 4:**
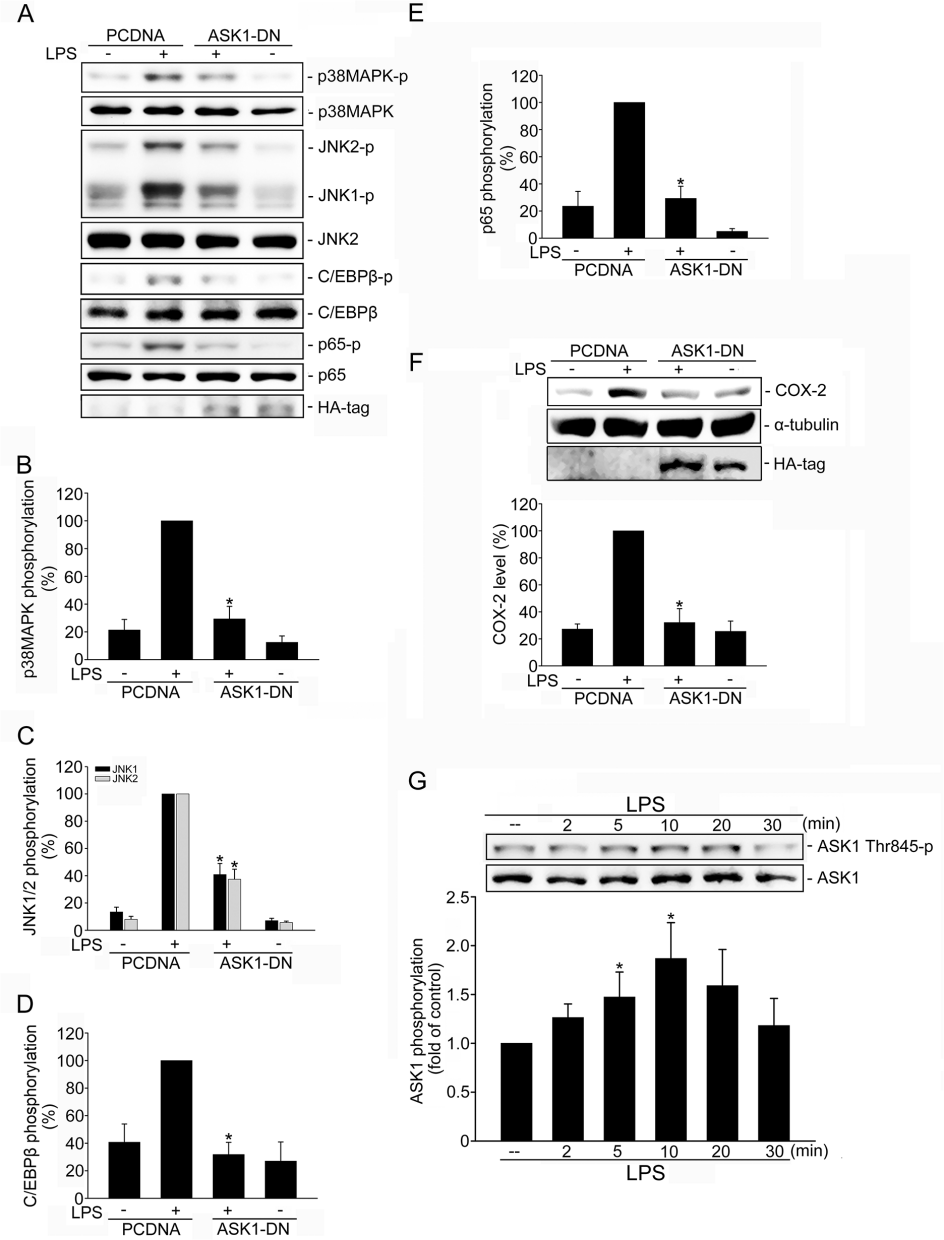
Involvement of ASK1 in LPS-induced p38MAPK, JNK1/2, C/EBPß and p65 phosphorylation and COX-2 expression in SV-LECs. Cells were transiently transfected with pcDNA or HA tagged ASK1-DN (HA-ASK1-DN) for 48 h followed by the treatment with LPS (3 mg/ml) for another 20 min. Phosphorylation status of p38MAPK, JNK1/2, C/EBPβ and p65 were then determined by immunoblotting. Figures shown in **(A)** are representative of at least four independent experiments with similar results. The compiled results of p38MAPK **(B)**, JNK1/2 **(C)**, C/EBPβ **(D)** and p65 **(E)** phosphorylations are shown at the bottom of the chart. Each column represents the mean ± S.E.M.. of four independent experiments. *p < 0.05, compared with the pcDNA group treated with LPS alone. **F.** Cells were transiently transfected with pcDNA or HA-ASK1-DN for 48 h followed by the treatment with LPS (3 mg/ml) for another 24 h. COX-2 level was then determined by immunoblotting. Each column represents the mean ± S.E.M.. of seven independent experiments. *p < 0.05, compared with the pcDNA group treated with LPS alone. **G.** Cells were treated with LPS (3 μg/ml) for the indicated time periods. Cells were then harvested and ASK1 Thr845 phosphorylation was determined by immunoblotting. Compiled results are shown at the bottom of the chart. Each column represents the mean ± S.E.M. of seven independent experiments. * p<0.05, compared with the control group.

We next examined whether the activation of ASK1, a critical upstream activator of p38MAPK and JNK1/2, contributes to LPS-induced COX-2 expression. Transfection of SV-LECs with ASK1 dominant negative mutant (ASK1-DN) significantly reduced LPS-induced p38MAPK (Fig 4B), JNK1/2 (Fig 4C), C/EBPß (Fig 4D) and p65 (Fig 4E) phosphorylation. ASK1-DN also suppressed COX-2 expression in LPS-stimulated SV-LECs (Fig 4F). Moreover, treatment of SV-LECs with LPS resulted in a time-dependent increase in ASK1 Thr845 phosphorylation (Fig 4G). Together these results suggest that ASK1 contributes to LPS’s actions in inducing COX-2 expression in SV-LECs.

### Involvement of PP2A in LPS-induced COX-2 expression in SV-LECs

**Fig 5:**
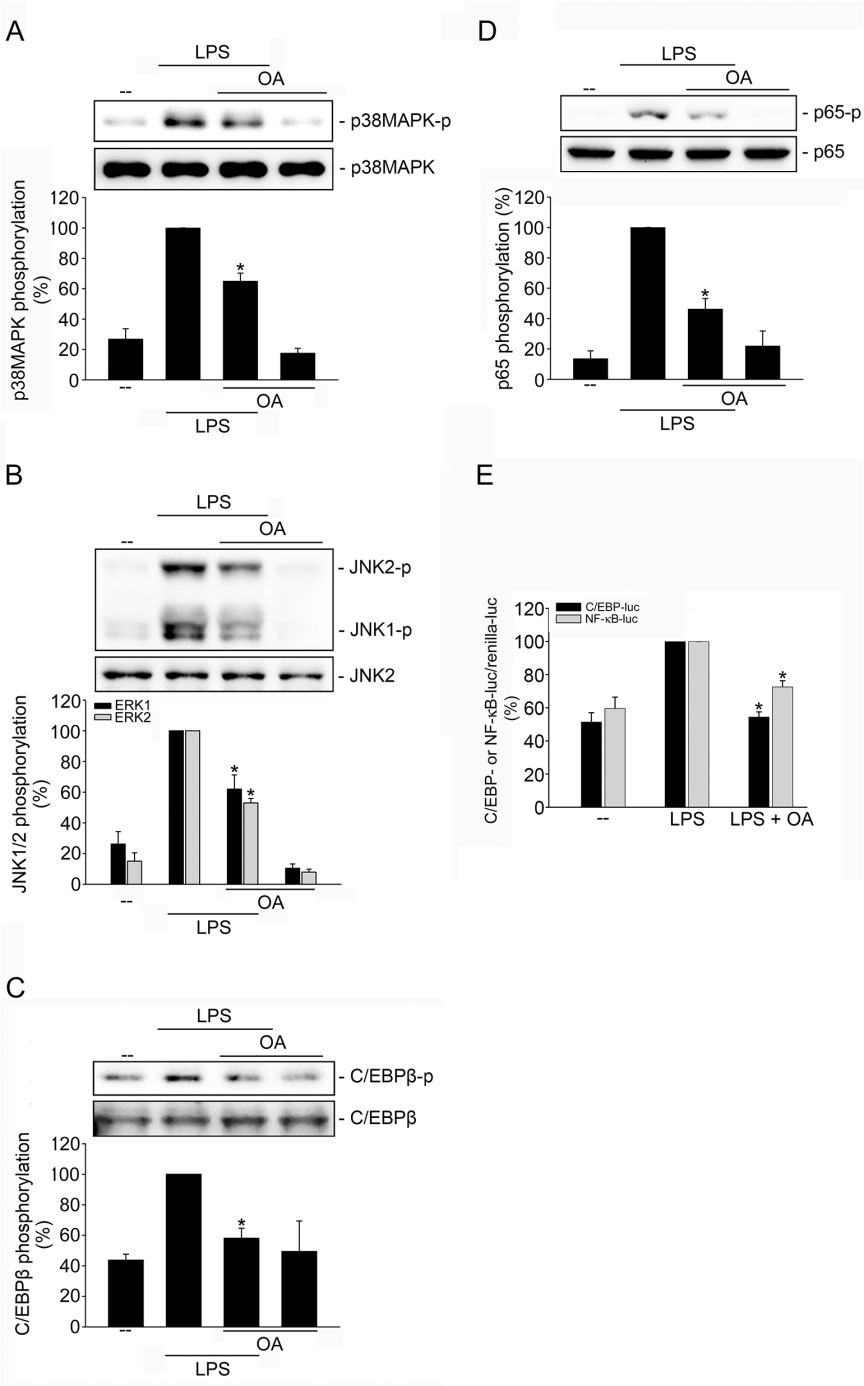
PP2A contributed to LPS-induced p38MAPK, JNK1/2, C/EBPß and p65 phosphorylation in SV-LECs. Cells were pretreated with a PP2A inhibitor okadaic acid (OA) (1 nM) for 30 min followed by treatment with LPS (3 μg/ml) for another 20 min. Phosphorylation status of p38MAPK **(A)**, JNK1/2 **(B)**, C/EBPβ **(C)** and p65 **(D)** were then determined by immunoblotting. The compiled results are shown at the bottom of the chart. Each column represents the mean ± S.E.M. of at least four independent experiments. *p < 0.05, compared with the group treated with LPS alone. **E.** Cells were transiently transfected with C/EBP-luc or NFκB-luc and renilla-luc for 48 h. After transfection, cells were pretreated with okadaic acid (OA) (1 nM) for 30 min followed by treatment with LPS (3 μg/ml) for another 24 h. Luciferase activity was then determined as described in the “Materials and Methods” section. Data represent the mean ± S.E.M. of six independent experiments performed in duplicate. * p<0.05, compared to the group treated with LPS alone.

**Fig 6:**
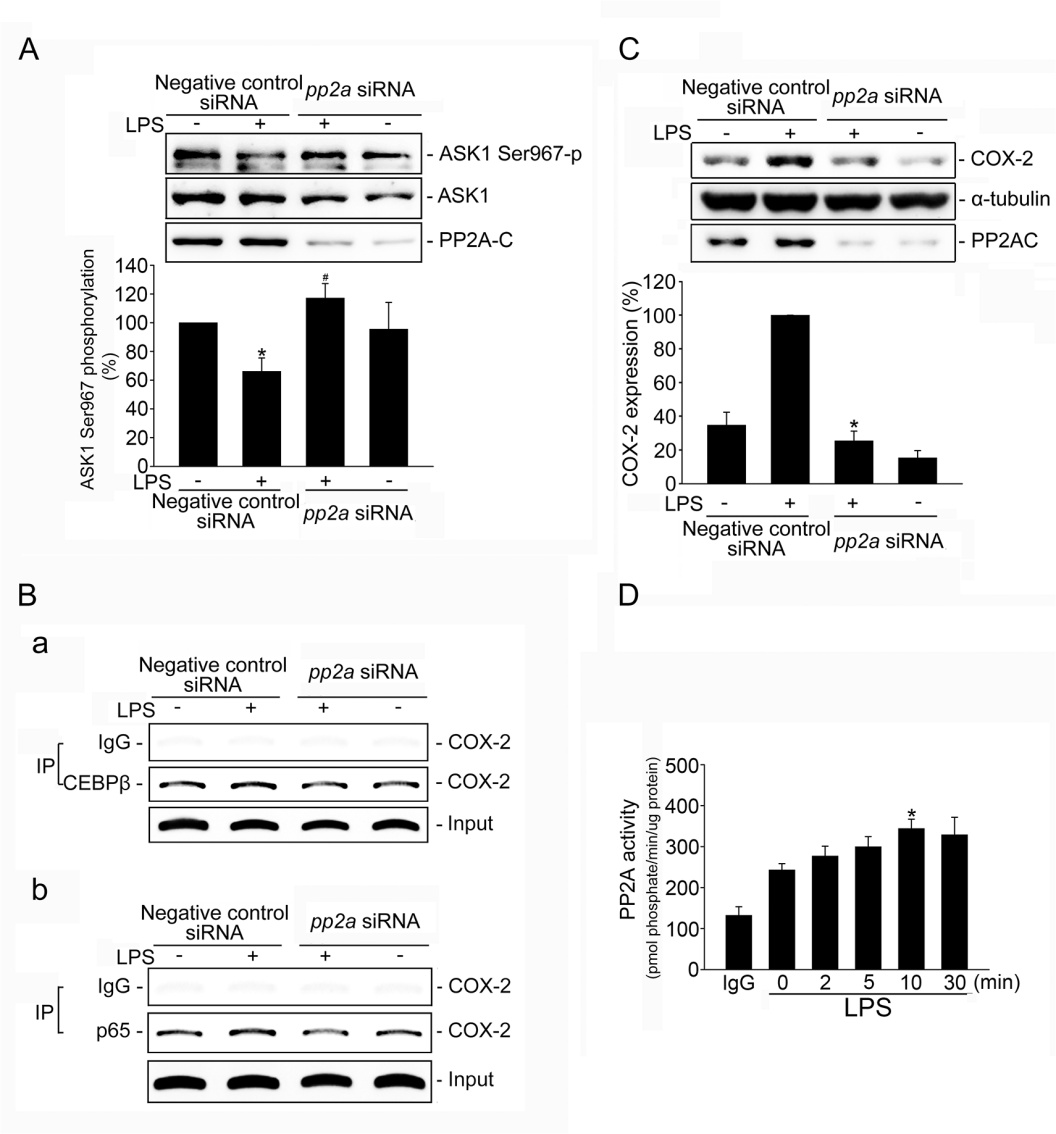
PP2A contributed to LPS-induced ASK1 Ser967 dephosphorylation and COX-2 expression in SV-LECs. Cells were transiently transfected with negative control siRNA or *pp2a* siRNA for 48 h followed by the treatment with LPS (3 μg/ml) for another 10 min. Phosphorylation status of ASK1 Ser976 was then determined by immunoblotting. The compiled results are shown at the bottom of the chart. Each column represents the mean ± S.E.M. of five independent experiments. *p < 0.05, compared with the negative control siRNA group treated with LPS alone. **B.** After transfection as described in (**A**), cells were treated with LPS (3 μg/ml) for another 2 h and the ChIP assay was performed as described in the ‘‘Materials and methods” section. Typical traces representative of three independent experiments with similar results are shown. **C.** After transfection as described in (**A**), cells were treated with LPS (3 μg/ml) for another 24 h. cells The COX-2 level was assessed by immunoblotting. The compiled results are shown at the bottom of the chart. Each column represents the mean ± S.E.M. of five independent experiments. *p < 0.05, compared with the negative control siRNA group treated with LPS alone. **D.** Cells were treated with LPS (3 μg/ml) for indicated time periods. Cells were then harvested for PP2A phosphatase activity analysis as described in the “Materials and Methods” section. Data represent the mean ± S.E.M. of four independent experiments. * p< 0.05, compared with the control group.

Several studies have highlighted PP2A’s role in regulating inflammatory responses [29, 30]. We reported previously that PP2A activates p38MAPK [31, 38] and JNK1/2 [30]. We therefore sought to explore whether PP2A is involved in LPS-induced p38MAPK or JNK activation. Similar to previous findings, okadaic acid, a selective PP2A inhibitor, reduced p38MAPK (Fig 5A) and JNK1/2 (Fig 5B) phosphorylation in LPS-stimulated SV-LECs. In addition, okadaic acid was effective in suppressing LPS-induced C/EBPß (Fig 5C) and p65 (Fig 5D) phosphorylation, as well as NFκB- or C/EBP-luciferase activities (Fig 5E). Phosphorylation of the ASK1 Ser967 residue increases ASK1 binding to the inhibitory 14-3-3 protein. ASK1 Ser967 de-phosphorylation leads to the dissociation of ASK1-14-3-3 complex and ASK1 activation [31, 39]. To ascertain the linkage between PP2A and ASK1 downstream of LPS, we next examined the effect of *pp2a* siRNA oligonucleotide on the phosphorylation status of ASK1 Ser967. Our experiments demonstrated that *pp2a* siRNA suppressed the basal level of PP2A catalytic subunit (PP2A-C) and restored LPS-decreased ASK1 Ser967 phosphorylation in SV-LECs (Fig 6A). Results from ChIP analysis showed that *pp2a* siRNA reduced the LPS’ effect on increasing C/EBPß (Fig 6Ba) and p65 (Fig 6Bb) binding to the COX-2 promoter region. Transfection of SV-LECs with *pp2a* siRNA also significantly reduced COX-2 expression in LPS-stimulated SV-LECs (Fig 6C). Furthermore, LPS caused an increase in PP2A activities in SV-LECs (Fig 6D). Taken together, these results suggest that PP2A mediates LPS-induced ASK1 activation and COX-2 expression in SV-LECs.

## Discussion

Lymphatic vasculature, instead of serving merely as a passive route for circulating immune cells, seems to be involved in the induction and the resolution of inflammation [1–3]. It undergoes substantial changes in experimental inflammatory animal models [5–7] and in human inflammatory diseases [4]. Although growing evidence shows that lymphatic vasculature is closely connected to inflammatory processes, little is known about the underlying mechanisms by which lymphatic endothelium responds to inflammatory stimuli. The limitations of studies on the signaling pathways in LECs are the difficulties in the isolation and propagation of LECs from different organs [40, 41]. We thus selected a ‘‘conditionally immortalized” line of murine LECs (SV-LECs) that express SV40 large T and retain their ‘lymphatic’ endothelial characteristics after repeated passages [33, 34]. In this study, we demonstrate that PP2A mediates COX-2 expression in SV-LECs exposed to, LPS, an inflammatory stimulus. The underlying mechanisms involve the activation of ASK1-JNK1/2 and/or ASK1-p38MAPK signaling cascade and subsequent NF-κB and C/EBPβ activation, which ultimately induces COX-2 expression in SV-LECs.

Similar to most mechanistic studies regarding cellular responses to endotoxin, we used relatively high concentration of LPS (1–3μg/ml) to explore the signaling mechanisms involved in COX-2 induction in SV-LECs. It is possible that results derived from these studies might not provide accurate depiction of biological activity of LPS, given that patients with critical illnesses such as sepsis have elevated plasma LPS levels up to 10 ng/ml [42, 43]. However, we noted that LPS at concentration as low as 10 ng/ml is capable of inducing COX-2 expression in SV-LECs. LPS at 10 ng/ml also caused increases in ERK1/2, p38MAPK and JNK1/2 phosphorylations in SV-LECs (S2 Fig). These observations suggest that SV-LECs respond to LPS at physiological and clinically relevant concentrations. Moreover, Maitra U. et al [44] recently demonstrated that low-dose LPS (50 pg/ml) exerts an opposing effects in modulating key negative regulators in TLR4 signaling as compared with LPS at the dose of 100 ng/ml in macrophages. Whether similar phenomenon could be observed in SV-LECs in response to LPS need to be further investigated.

COX-2 was reported to regulate vascular endothelial functions and play an important role in inflammation [11, 12]. Elevation of COX-2 levels was observed in immune cells, cerebral microvascular endothelial cells and vascular endothelial cells in response to many inflammatory stimuli including LPS [14, 30, 45]. Although LECs are terminally differentiated cells distinct from blood vascular endothelial cells [46, 47], we noted in this study that LPS also induced COX-2 expression in LECs, the mechanism of which was explored in this study. In addition, alterations in lymphatic function are associated with inflammation. Cromer W. E. et al [48] recently demonstrated that LECs increase their permeability in response to LPS, leading to the loss of barrier function of the LEC monolayers *in vitro*. Whether the integrity of the lymphatic endothelial barrier is altered in response to LPS *in vivo* and the role of COX-2 in regulating lymphatic barrier function needs to be further investigated.

ASK1 plays a critical role in regulating diverse cellular processes by stimulating p38MAPK or JNK1/2 signaling [24, 26–28]. Activation of MAPKs is required for COX-2 induction in human vascular endothelial cells exposed to LPS per previous report [45], but whether the ASK1-JNK1/2 or ASK1-p38MAPK signaling cascade participates in LPS-induced COX-2 expression in LECs was unclear. In this study, we noted that LPS caused activations of ASK1 and MAPKs. ASK1-DN suppressed LPS-induced p38MAPK and JNK1/2 activations. ASK1-DN and inhibitors of MAPKs signaling also markedly suppressed COX-2 expression in LPS-stimulated SV-LECs. These results suggest that LPS activates ASK1 to cause p38MAPK and JNK1/2 activation, leading to COX-2 expression in SV-LECs. We also noted that ERK1/2 activation, which is not induced by ASK1 [24], also contributed to COX-2 induction in LPS-stimulated SV-LECs. Further investigations are needed to explore the mechanisms of LPS in activating ERK1/2 signaling in SV-LECs.

Phosphorylation of ASK1’s Thr845 residue, one of the autophosphorylation sites, is essential for its activation [49]. In contrast, ASK1 Ser967 phosphorylation promotes ASK1 binding to inhibitory 14-3-3 proteins, resulting in suppression of ASK1 activity [50]. Recent studies suggest that PP2A is responsible for ASK1 Ser967 dephosphorylation and ASK1 activation in COS7 fibroblasts [51], cerebral endothelial cells [31] and macrophages [30]. In agreement with these observations, we found that LPS increased PP2A activity, and that *pp2a* siRNA restored PGN-induced ASK1 Ser967 dephosphorylation and COX-2 expression. LPS also caused ASK1 Thr845 phosphorylation. These findings suggest that PP2A plays a pivotal role in ASK1 Ser967 dephosphorylation and subsequent signaling events in SV-LECs after LPS exposure. The precise mechanism involved in LPS-induced PP2A activation in SV-LECs needs to be investigated further. PP2A is a member of ceramide-activated protein phosphatases family and is activated by ceramide. Cellular ceramide increases in response to stress stimuli [52]. Ceramide formation may result from sphingomyelin hydrolysis by acidic or neutral sphingomyelinase (nSMase) [53]. We previously demonstrated that nSMase mediates PP2A activation in macrophages [30] and vascular smooth muscle cells [29]. We also noted that nSMase blockade significantly inhibited LPS-induced COX-2 expression in SV-LECs (unpublished data). These findings raise the possibility that the nSMase-ceramide cascade mediates LPS-induced PP2A activation and COX-2 expression in SV-LECs.

Transcription factor NF-κB plays a causal role in the pathogenesis of various vascular diseases marked by abnormal proliferation and inflammation [54–56]. Similar to previous reports [34, 57], we noted that p38MAPK and JNK1/2 contribute to LPS-induced NF-κB and C/EBPβ activation in SV-LECs. However, the mechanisms of this activation, and whether this leads to lymphatic-specific genes expression, as suggested in another study [34], remain incompletely understood. In keeping with previous observations, we demonstrated that LPS activates NF-κB and C/EBPβ resulting in COX2 induction in SV-LECs as well as in cerebral endothelial cells [14]. In addition to NF-κB and C/EBPβ, previous study has indicated that transcription factor activator protein-1 (AP-1) also contributes to the induction of COX-2 [58]. It is established that activation of p38MAPK or JNK1/2 leads to AP-1 activation [59]. We also found that blockade of MAPKs signaling reduces LPS’s effects on COX-2 expression in SV-LECs. These findings suggest that LPS-induced COX-2 expression is attributable to not only NF-κB and C/EBPβ, but also AP-1 in SV-LECs. Moreover, C/EBPβ has been shown to interact with NF-κB family members [60, 61] or AP-1 [62]. Additional works are needed to investigate whether C/EBPβ cooperates with NF-κB, AP-1 or other transcription factors in the induction of COX-2 in LPS-stimulated SV-LECs. Furthermore, cellular COX-2 level may be regulated through posttranslational mechanisms [63]. Dean et al [64] reported that p38MAPK signaling contributes to the increase in COX-2 mRNA stability in LPS-stimulated monocytes. It is likely that COX-2 induction by LPS may not only involve transcriptional, but also post-transcriptional and/or post-translational mechanisms.

In conclusion, we demonstrated in this study that PP2A plays a causal role in LPS activation of the ASK1-p38MAPK and/or JNK1/2-C/EBPβ and/or NF-κB signaling cascade, leading to COX-2 expression in SV-LECs. The present study delineates, at least in part, the signaling pathways involved in LPS-induced COX-2 expression in SV-LECs. A better understanding of these signaling mechanisms may help facilitate development of therapeutic strategies to reduce lymphatic inflammation caused by Gram-negative organisms.

## Supporting Information

S1 Fig SV-LECs express lymphatic endothelial markers. The extent of *VEGFR-3*, *LYVE-1* and *Prox-1* mRNA was determined by an RT-PCR assay as described in the “Materials and methods” section. Typical traces representative of three independent experiments with similar results are shown. MCEC: murine cerebral endothelial cell.
(PDF)

S2 Fig LPS induced ERK1/2, p38MAPK and JNK1/2 phosphorylation in SV-LECs. SV-LECs were treated with LPS (10 ng/ml) for 10 min. Phosphorylation status of ERK1/2, JNK1/2 and p38MAPK were then determined by immunoblotting. Figures shown in (A) are representative of at least four independent experiments with similar results. The compiled results of ERK1/2 (B), JNK1/2 (C), and p38MAPK (D) phosphorylations are shown. Each column represents the mean ± S.E.M. of four independent experiments. *p < 0.05, compared with the control group.
(PDF)
