# Supplementary material for: Protein Phosphatase 2A in Lipopolysaccharide-Induced Cyclooxygenase-2 Expression in Murine Lymphatic Endothelial Cells
Source: PLoS One. 2015 Aug 28;10(8):e0137177. doi: 10.1371/journal.pone.0137177 (PMC4552685; doi:10.1371/journal.pone.0137177)
Supplement: S1 Fig — The extent of VEGFR-3, LYVE-1 and Prox-1 mRNA was determined by an RT-PCR assay as described in the “Materials and methods” section. Typical traces representative of three independent experiments with similar results are shown. MCEC: murine cerebral endothelial cell. (PDF) [file pone.0137177.s001.pdf]

S1 Fig.

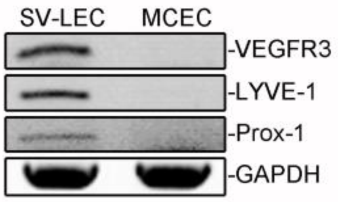

S1 Fig. SV-LECs express lymphatic endothelial markers

The extent of *VEGFR-3, LYVE-1* and *Prox-1* mRNA was determined by an RT-PCR assay as described in the “Materials and methods” section. Typical traces representative of three independent experiments with similar results are shown. MCEC: murine cerebral endothelial cell.
