# Supplementary material for: Protein Phosphatase 2A in Lipopolysaccharide-Induced Cyclooxygenase-2 Expression in Murine Lymphatic Endothelial Cells
Source: PLoS One. 2015 Aug 28;10(8):e0137177. doi: 10.1371/journal.pone.0137177 (PMC4552685; doi:10.1371/journal.pone.0137177)
Supplement: S2 Fig — SV-LECs were treated with LPS (10 ng/ml) for 10 min. Phosphorylation status of ERK1/2, JNK1/2 and p38MAPK were then determined by immunoblotting. Figures shown in (A) are representative of at least four independent experiments with similar results. The compiled results of ERK1/2 (B), JNK1/2 (C), and p38MAPK (D) phosphorylations are shown. Each column represents the mean ± S.E.M. of four independent experiments. *p < 0.05, compared with the control group. (PDF) [file pone.0137177.s002.pdf]

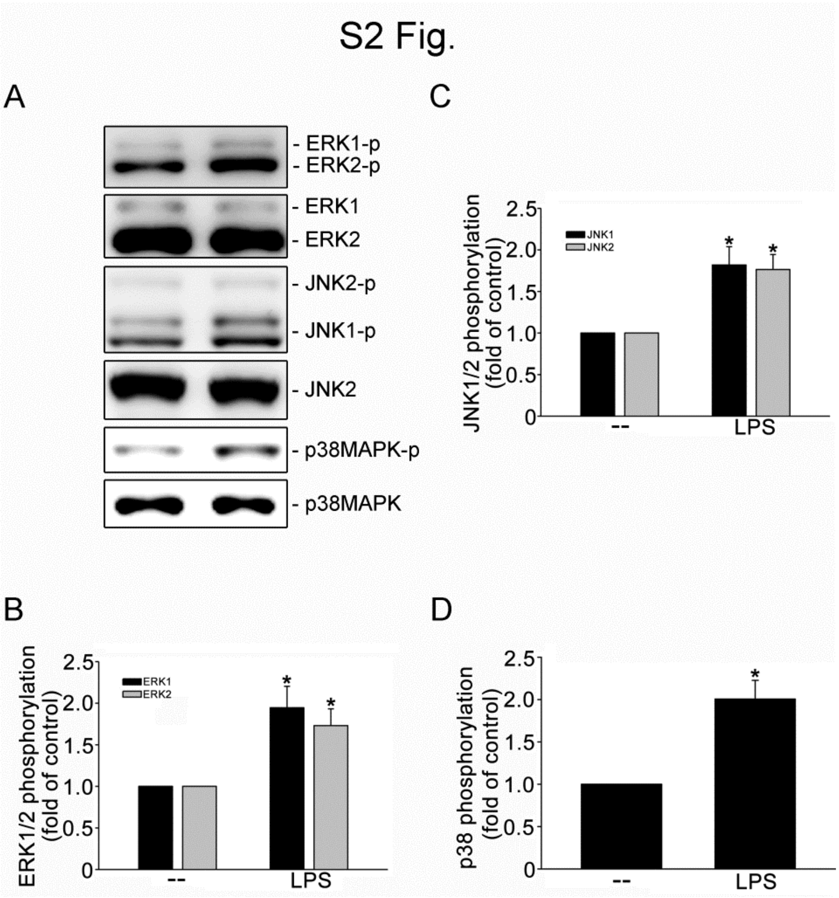

S2 Fig. LPS induced ERK1/2, p38MAPK and JNK1/2 phosphorylation in SV-LECs

SV-LECs were treated with LPS (10 ng/ml) for 10 min. Phosphorylation status of ERK1/2, JNK1/2 and p38MAPK were then determined by immunoblotting. Figures shown in (A) are representative of at least four independent experiments with similar results. The compiled results of ERK1/2 (B), JNK1/2 (C), and p38MAPK (D) phosphorylations are shown. Each column represents the mean ± S.E.M. of four independent experiments. *p < 0.05, compared with the control group
